# Evaluation of clinical characteristics and risk factors associated with Chlamydia psittaci infection based on metagenomic next-generation sequencing

**DOI:** 10.1186/s12866-024-03236-1

**Published:** 2024-03-13

**Authors:** Lei Yuan, Qiang Chen, Xin Yu Zhu, Lan Min Lai, Rui Zhao, Yang Liu

**Affiliations:** Department of Clinical laboratory, The First Affiliated Hospital of Nanchang University of Nanchang University, Nanchang University, No.17, YongWaiZhengStreet, Nanchang, 330006 China

**Keywords:** Metagenomic next-generation sequencing, *Chlamydia psittaci*, Pneumonia, Diagnosis, Therapeutic, Lower respiratory tract microbiota

## Abstract

**Introduction:**

Psittacosis is a zoonosis caused by *Chlamydia psittaci*, the clinical manifestations of Psittacosis range from mild illness to fulminant severe pneumonia with multiple organ failure. This study aimed to evaluate the clinical characteristics of *Chlamydia psittaci* infection diagnosed based on metagenomic next-generation sequencing(mNGS), as well as the risk factors affecting the progress of *Chlamydia psittaci* infection, in order to improve the effect of therapeutics.

**Methods:**

We retrospectively analyzed the clinical data of patients infected with *chlamydia psittaci* in the First Affiliated Hospital of Nanchang University from January 2021 to December 2021. The patient’s past medical history, clinical manifestations, laboratory examinations, chest CT results, treatment status, and prognosis data were collected. we also investigated both the pathogenic profile characteristics and the lower respiratory tract microbiota of patients with C*hlamydia psittaci* pneumonia using mNGS.

**Results:**

All cases of *Chlamydia psittaci* in our research have been confirmed by mNGS. Among 46 cases of *Chlamydia psittaci* pneumonia, Poultry exposure was reported in 35 cases. In severe cases of *Chlamydia psittaci* pneumonia, Neutrophils, Procalcitonin (PCT), Lactate Dehydrogenase (LDH), Hydroxybutyrate Dehydrogenase (HBDH), Creatine Kinase Isoenzymes-B (CK-MB) and D-Dimer levels were remarkably higher than that of non-severe cases, except for lymphocytes (all *P* < 0.05). Chest CT scans showed Bilateral (77.8%), multiple lobar lungs (85.2%), pleural effusions (44.4%) involvement in those suffering from severe *Chlamydia psittaci* pneumonia, whereas its incidence was 0%, 21.1% and 10.5% in non-severe patients, respectively (*P* < 0.05). Multivariate analysis revealed that higher lymphocyte concentrations (OR 0.836, 95% CI 0.714–0.962, *P* = 0.041) were the only protective factor for survival. mNGS results indicated that 41.3% of patients (19/46) had suspected coinfections with a coinfection rate of 84.2% (16/19) in the severe group, much higher than that in the non severe group (*p <* 0.05). No significantly different profiles of lower respiratory tract microbiota diversity were found between non severe group and severe group.

**Conclusion:**

A history of poultry exposure in patients can serve as an important basis for diagnosing *Chlamydia psittaci* pneumonia, and patients with severe *Chlamydia psittaci* pneumonia are more likely to develop elevated inflammatory biomarkers as well as elevated cardiac markers. Higher lymphocyte concentrations are protective factors associated with severe *C. psittaci* pneumonia. The higher proportion of patients with coinfections in our study supports the use of mNGS for comprehensive early detection of respiratory infections in patients with *C. psittaci* pneumonia.

## Introduction

Community acquired pneumonia (CAP) is one of the most universal infectious diseases in intensive care unit(ICU), with a mortality rate of 30–50% [1], *Chlamydia Psittacosis*(*C. psittaci*), as the pathogen of CAP, accounting for 0-6.7% of the CAP infection [2]. *C. psittaci* is an obligate intracellular bacterium that grows in eukaryotic cells and can cause zoonotic disease psittacosis, which can lead to widespread infection in humans and animals, causing serious economic losses to the breeding industry, and posing a huge menace to human public health security [3, 4]. Humans mainly cause infection through the respiratory system. Bacteria that enter the human body diffuse into the reticuloendothelial system through the bloodstream, causing lung lesions and potentially affecting other organ systems,, including the liver, spleen, meninges and central nervous system [5]. The clinical manifestations of *C. psittaci* pneumonia range from mild symptoms to severe pneumonia. The main symptoms are respiratory infections, manifested as non-specific influenza-like symptoms such as fever, chills, sore throat and headache, as well as fatigue and muscle pain. Other non-specific symptoms include skin rash, vomiting, diarrhea [6, 7].

In recent years, severe *C. psittaci* pneumonia has been reported more frequently in clinical practice [8]. Early diagnosis of *C. psittaci* infection is one of the critical approachs for its prevention and control, and accurate detection results play an important role in controlling the spread of pathogens. However, Early diagnosis of *C. psittaci* pneumonia is challenging because the disease is characterized by non-specific symptoms [9], The traditional detection methods for *C. psittaci* include pathogen detection, serological detection, and molecular biology detection, among others. Culture is still the gold standard for the diagnosis of *C. psittaci* infection, but the diagnosis and treatment of *C. psittaci* infection will be delayed due to its strict nutritional requirements and long culture time. Besides, serological detection techniques such as immunofluorescence and complement binding assay have drawbacks such as long detection cycles, insufficient specificity or sensitivity [10]. With the development of detection technology, while ensuring specificity and sensitivity, researchers have further increased their requirements for detection speed. Molecular biology detection technology stands out because it is more suitable for laboratory testing and epidemiological investigations.

Metagenomic next-generation sequencing (mNGS) can quickly detect bacteria, viruses, fungi, protozoa, and other multicellular eukaryotic pathogens [11], achieving rapid screeningwithout specific amplification, which is helpful to identify pathogens in time to customize specific anti infective treatment [12], in addition, mNGS also plays a good auxiliary role in the clinical diagnosis of unexplained pneumonia, especially suitable for the diagnosis of difficult and complicated cases. In view of this, we conducted a retrospective study to investigate the clinical characteristics of severe *C. psittaci* pneumonia diagnosed by mNGS and identify the risk factors for poor prognosis of *C. psittaci* infection, in order to provide clinical suggestions and new insights into the pathogenesis of infectious pneumonia caused by *C. psittaci*.

## Materials and methods

### Patients and specimens

Retrospective analysis was conducted in patients admitted to the First Affiliated Hospital of Nanchang University in 2021 with *C. psittaci* infection. For each case, data on prodromal symptoms, disease severity, dynamics, and comprehensive computer tomography and detection indicators were extracted from electronic medical records. Other data on therapeutic drugs and outcomes were also collected. In our study, pathogen diagnosis was judged by clinicians according to imaging, clinical features and clinical examinations.

### Diagnostic criteria

The diagnostic criteria for *C. psittaci* pneumonia was defined as follows: (I) Individuals fulfilling the diagnostic criteria for CAP according to Chinese adult guidelines for diagnosis and treatment of community-acquired pneumonia [13]. (II) The existence of specific *C. psittaci* gene fragments in samples detected by mNGS. (III) The results are consistent with the diagnosis of clinicians. Diagnostic criteria for severe pneumonia are as follows [13]: Severe pneumonia can be diagnosed if one or more of the following signs occur. (1) Disturbance of consciousness; (2) Respiratory rate ≥ 30 times/min; (3) PaO2 < 60mmHg; PaO2/FiO2 < 300, requiring mechanical ventilation; (4) Arterial systolic blood pressure < 90mmHg; (5) Septic shock; (6) The X-ray chest film showed bilateral or multiple pulmonary lobe involvement, or the lesion expanded ≥ 50% within 48 h of Hospital; (7) Oliguria: urine volume < 20 ml/h, or < 80 ml/4 h, or complicated with acute renal failure, requiring dialysis treatment.

### Metagenomic next-generation sequencing and analysis

#### Sample collection

34 samples of alveolar lavage fluid, 5 sampless of sputum, and 7 samples of peripheral blood samples were collected for analysis. Blood samples were centrifuged at 1600 g for 10 min in order to separate plasma supernatant for extracting DNA. The sample was stored at -80 ° C for long-term storage.

#### Nucleic acid extraction

Nucleic acid extracted from all samples including the negative controls (water and extraction buffer) were acquired using the TIANamp Micro DNA Kit (TIANGEN Biotech, Beijing, China) in accordance with the manufacturer’s manual. Qubit dsDNA HS Assay Kit (ThermoFisher Scientific) were used for nucleic acid concentration determination according to the manufacturer’s manual.

### Library construction and sequencing

Libraries were constructed by using the Nextera XT kit (Illumina) following the manufacturer manual. Illumina NextSeq-550Dx sequencer was employed for the sequencing reaction to gain the sample sequence information in the sample. The sequencing depth of each sample ≥ 20 million reads was sequenced using a 75-cycle single-end sequencing strategy.

### Bioinformatics analysis

Bcl2fastq v2.20.0.422 with default parameters was applied to demultiplex the primary sequencing output. For quality control adapter contamination and low-quality and low-complexity reads, raw reads and adapter removal were filtered by fastp (v0.19.5) [14] and Komplexity v0.3.6 [15]. Samples with < 10 million reads after QC are treated as unqualified and need to be re-sampled and tested. Reads that were mapped to the human reference assembly GRCh38 were removed with Bowtie2 v2.3.4.3 [16].Then, reads were aligned to the microorganism database consisting of approximately 12,000 genomes with SNAP v1.0 beta.18 [17] as previously described [18]. The mapped reads were classified based on the NCBI RefSeq genome database, or the NCBI GenBank genome database (http://ftp.ncbi.nlm.nih.gov/genomes/) was selected for each species. After filtering low-complexity reads [19], we counted the species or genus abundance with Perl scripts.

### Reporting

The following were criteria for positive results of mNGS [20]:

1. For intracellular bacterium (*C. psittaci*, *Mycobacterium spp*,ect) and parasites, due to the difficulty of DNA extraction and low possibility for contamination, the result was considered positive if the number of detected sequences had the reads per million (RPM) ≥ 1.
2. For opportunistic pathogens, fungi and virus, the result was considered positive if the number of detected sequences were found > 5 folds in samples than in controls.

### Statistical analysis

All statistical analyses were conducted using SPSS 26.0 software, with counting data described in terms of frequency and component ratio. Continuous variables of normal distribution are expressed as means ± SD(standard), or as medians (25th, 75th percentiles) for non-normal distribution. Continuous variables were compared by Mann-Whitney U-test or t-test, and categorical variables were analyzed using the Fisher’s exact test. Using multivariate logistic regression analysis to identify risk factors associated with severe *C. psittaci* pneumonia. *P* < 0.05 in univariate analysis was considered for the multivariate model. *P* < 0.05 was considered statistically significant for the difference.

## Results

### General characteristics of the enrolled cohort

Among the 46 patients, 33 were male and 13 were female; The age range is 20–86 years old; The length of hospitalization time ranged from 3 to 52 days, and 35 patients had a clear history of bird contact; The incidence was mainly concentrated in autumn and winter (from September of that year to February of the next year). The most common clinical symptoms included fever (82.6%, 38/46), cough (37.0%, 17/46), expectoration (26.10%, 12/46), chest tightness (8.70%, 4/46), dyspnea (8.70%, 4/46), limb fatigue (10.87%, 5/46), etc. Past medical history included hypertension (3.04%, 14/46), diabetes (10.87%, 5/46), cirrhosis (4.34%, 2/46), cerebral infarction (6.52%, 3/46), nephrotic syndrome (2.17%, 1/46), myelodysplastic syndrome (2.17%, 1/46), coronary heart disease (4.34%, 2/46), nodulosis (2.17%, 1/46), gastric ulcer (2.17%, 1/46). Clinical manifestations of the enrolled patients are shown in Table 1.

**Table 1:** Clinical manifestations of patients with *C. psittaci* pneumonia

Characteristics	*C. psittaci* pneumonia (*n* = 46)	Non severe *C. psittaci* pneumonia (*n* = 19)	Severe *C. psittaci* pneumonia (*n* = 27)	P value
**Demographics**				
Male/female	33/13	12/7	21/6	0.2782
Age, median (range, years)	61.7 ± 14.5	63.6 ± 14.2	60.3 ± 19.5	0.4749
Length of stay (days)	16.6 ± 10.8	12.5 ± 9.7	15.1 ± 12.3	0.0563
**Season of admission, n**				0.4110
Spring	8	2	6	
Summer	6	3	3	
Autumn	12	5	7	
Winter	20	9	11	
**Clinical manifestations, n**				
Fever	38	17	21	0.4395
Cough	17	5	12	0.2352
Expectoration	12	4	8	0.7346
Chest tightness	4	1	3	0.6322
Dyspnoea	4	0	4	0.1313
Hypodynamia	5	1	4	0.3870
Chills	1	0	1	1.0000
Headache	1	1	0	0.4419
Disturbance of consciousness	2	0	2	0.4950
**Underlying disease, n**				
Hypertension	14	4	10	0.3352
Diabetes	5	2	3	1.0000
Cirrhosis	2	1	1	1.0000
Cerebral infarction	3	2	1	0.5607
Nephrotic syndrome	1	0	1	1.0000
Myelodysplastic syndrome	2	0	2	0.5043
Coronary heart disease	2	1	1	1.0000
Nodulosis	1	0	1	1.0000
Gastric ulcer	1	0	1	1.0000

### Laboratory characteristics

All 25 patients with *C. psittaci* pneumonia had a total leukocytes count within the normal range, of which 17 had leukocytes exceeding 9.5 × 10^9^/L, 1 case below 3.5 × 10^9^/L.The number of neutrophils increased in some cases, the proportion of neutrophils in 26 cases was higher than normal, and 1 case was lower than the normal range. hs-CRP was detected in 42 patients and increased in almost all cases (40/41). 32 patients underwent Erythrocyte sedimentation rate(ESR), of which 31 were higher than the normal range. The PCT data of 42 patients showed that 20 cases were higher than normal, and 22 cases had PCT values within the normal range.All patients were tested for coagulation function, including D-D dimer, prothrombin time(PT), Activated partial thromboplastin time (APTT), FIB and thrombin time(TT). The more characteristic laboratory abnormalities are D-D dimer, PT and FIB. 76.1%, 73.9% and 95.7% of the cases increased respectively. The number of patients with elevated AST (73.8%, 31/42) was higher than that of patients with ALT (50%, 21/42). Among the patients who performed myocardial zymogram, LDH and CK increased in 82.9% and 35.9% respectively. Compared to patients with the non-severe infection, those with severe *C. psittaci* pneumonia exhibited significantly lower lymphocyte and higher Neutrophils, PCT, LDH, HBDH, CK-MB and D-Dimer levels(*p*<0.05). Laboratory results are shown in Table 2.

**Table 2:** Laboratory examination on admission of patients with *C. psittaci* pneumonia

Characteristics	*C. psittaci* pneumonia (*n* = 46)	Non severe *C. psittaci* pneumonia (*n* = 29)	Severe *C. psittaci* pneumonia (*n* = 27)	P value
WBC (3.5–9.5 × 10^9^ /L)	7.60(5.50, 9.88)	6.92(5.36, 9.24)	9.01(0.66,10.11)	0.0725
Neutrophils(1.8–6.3 × 10^9^ /L)	6.61(4.35, 9.28)	6.25(4.25, 8.06)	8.06(5.66, 9.76)	**0.035**
Lymphocyte (1.1–3.2 × 10^9^ /L)	0.65 ± 0.53	0.74 ± 0.51	0.48 ± 0.46	**0.0016**
CRP (0–8 mg/L)	184.61 ± 97.91	185.63 ± 95.55	201.55 ± 101.65	0.2531
ESR (0–20)	53.5(35.75, 72.5)	53.5(42, 68.5)	52(30, 72)	0.4727
PCT (0–0.5 ng/mL)	0.47(0.25, 2.42)	0.39(0.25, 1.87)	1.75(0.44, 4,32)	**0.001**
ALT (9–50 U/L)	50(25.5, 93.1)	54(27, 94.8)	54(31, 91.4)	0.4464
AST (15–40 U/L)	78.4(37.25, 133)	75(44, 121)	97(45, 168.5)	0.0786
LDH (120–250 U/L)	364.4(266.45,535.4)	327.2(216.63, 503.5)	449.35(296.08, 741)	**0.0473**
HBDH (72–182 U/L)	226.7(203.25, 438.2)	214.95(200.5, 438.2)	331.8(217, 592)	**0.0388**
CK (50–310 U/L)	172.9(83.45, 536)	174.45(90.475,485.93)	230.9(109.5, 1152.9)	0.0595
CK-MB(2–25U/L)	18.6(13.7, 34.4)	17.2(12.35, 25.23)	24(14.15, 49)	**0.0441**
IL-6 (0–11.09 pg/mL)	223.11 ± 406.95	106.69 ± 277.29	292.96 ± 395.44	0.1554
D-Dimer(0-0.55 mg/L)	2(0.61, 5.06)	1.46(0.59, 4.19)	2.83(0.94, 5.63)	**0.0137**
PT(9.8-12.1s)	12.9(11.98, 14.08)	12.9(12.2, 14)	12.9(12.3, 13.95)	0.4889
APTT(25.3-33.8s)	30.9(28.58, 34.85)	30.5(28.5, 33.3)	31(28.65, 37.9)	0.4752
FIB(1.8-3.5 g/L)	5.74(4.72, 6.38)	5.55(4.77, 6.17)	5.93(4.58, 6.51)	0.6472
TT(14-21s)	15.5(14.23, 16.63)	15.4(14.2, 16.7)	15.3(14.2, 16.3)	0.4481

### Radiological manifestations

All patients underwent computerized tomography(CT) of lung before admission. Most patients with psittacosis developed lobar pneumonia (37 cases, 80.4%), and the rest showed flake or strip-shaped high-density shadow; The lesions were unilateral in 25 cases (54.3%), including 10 cases on the left and 15 cases on the right; Bilateral 21 cases (45.6%); There were 17 cases of single leaf involvement, including 10 cases of upper leaf lesions, 7 cases of middle and lower leaf lesions and 29 cases of multiple leaf involvement. Patients in the severe group had more bilateral lung lesions(77.8%)than the non-severe group, pleural effusion(44.4%) and multiple lobar lesions(85.2%) were more observed in the severe group(*p*<0.05). The imaging features during admission are shown in Table 3.

**Table 3:** Radiological manifestations of patients with *C. psittaci* pneumonia

Characteristics	*C. psittaci* pneumonia (*n* = 46)	Non severe *C. psittaci* pneumonia (*n* = 19)	Severe *C. psittaci* pneumonia (*n* = 27)	P value
**Imaging, n**				
Scope of lesions				
Unilateral, Left	10	8	2	**< 0.01**
Unilateral, Right	15	11	4	
Bilateral	21	0	21	
Single lobe	19	15	4	**< 0.01**
Multiple lobar	27	4	23	
**Pleural effusions, n**				**0.0218**
No pleural effusions	32	17	15	
Unilateral	4	2	2	
Bilateral	10	0	10	

### Co-infection of *C. Psittaci* pneumonia patients with other pathogens based on mNGS

**Fig. 1:**
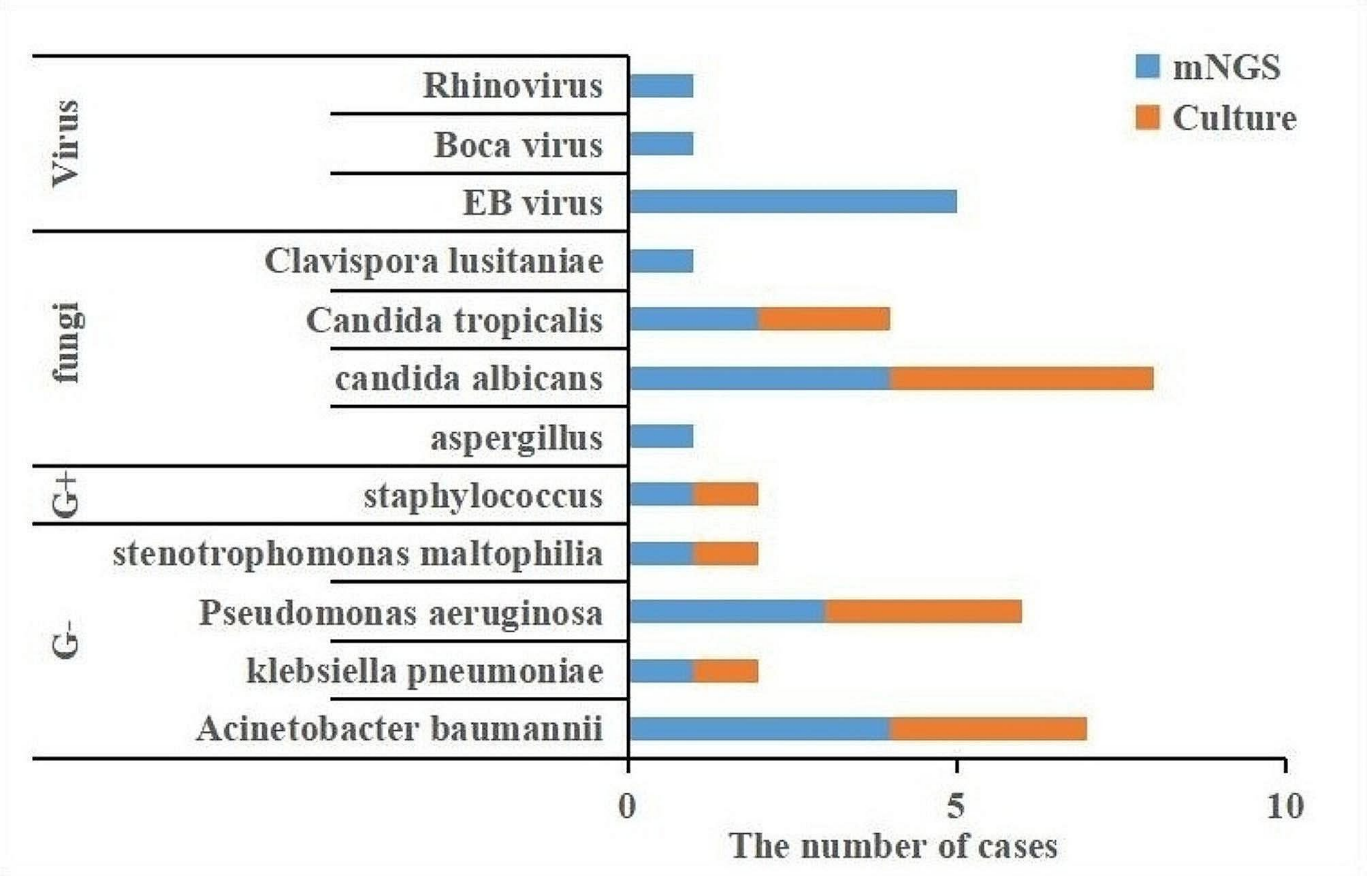
Distribution of pathogens identifed by mNGS(G+: Gram positive bacteria; G-: Gram negative bacteria)

In this study, clinical samples identified as *C. psittaci* infection using mNGS included 34 samples of BALF, 5 samples of sputum, and 7 samples of peripheral blood. Among 46 patients,41.3% (19/46) patients had mixed infection, including 7 patients with bacterial infection, 5 patients with viral infection and 3 patients with fungal infection. 2 cases of co infection with bacteria and fungi, and 2 cases of co infection with fungi and viruses. The comprehensive detection rates of bacteria, fungi and viruses were 19.56%(9/46), 15.21%(7/46) and 15.21%(7/46) respectively. mNGS yields a higher positive detection rate for pathogens compared with the culture method. mNGS results indicated that 41.3% of patients (19/46) had suspected coinfections with a coinfection rate of 84.2% (16/19) in the severe group. much higher than that in the non severe group(*p*<0.05). The most common bacterial co-infection is mainly related to Acinetobacter baumannii. The most common co-infection of virus and fungus were Candida albicans and EB virus(Figure. 1).

### Characterization of Lower respiratory tract microbiota in *C. Psittaci* pneumonia

**Fig. 2:**
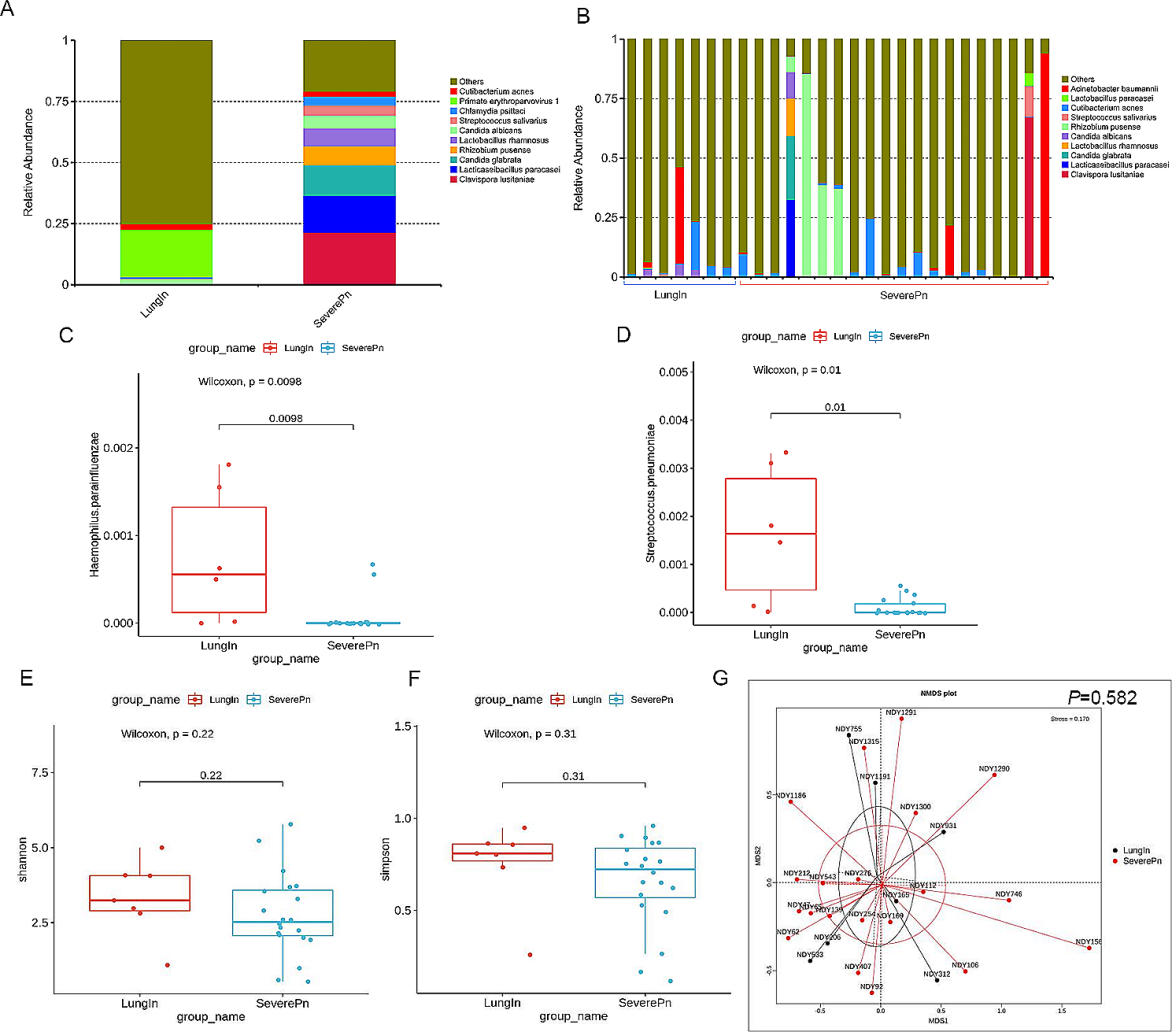
(**A**) Relative abundance of the 10 most abundant species in *C. psittaci* pneumonia (LungIn)and severe *C. psittaci* pneumonia(SeverePn). (**B**) Composition of microbiome in the different samples. (**C**-**D**) The abundance of *Haemophilus parainfluenzae* and *Streptococcus pneumoniae* in different groups. The boxes represent 25th -75th percentiles, the horizontal lines indicate the median, and the whiskers were drawn from the box to the extremes (values that were lower/greater than first/third quartile minus/plus 1.5 times the interquartile range were regarded as outliers). (**E**-**F**) Alpha diversity differences of lung microbiota communities at species level between groups (Shannon index and Simpson index). (**G**) Principal coordinate analysis plot(PCoA) of samples at the species level. Each dot represents one sample from each group, and the color of the points represents the group in which the sample is located

A series of previous studies have shown that imbalance of microbiota in the lung may be closely associated with the development of pulmonary(infectious) diseases. Therefore, we also investigated the effects of *C. psittaci* infection on the microbiota of the lower respiratory tract by analyzing the characteristics of the lung microbial communities detected by mNGS. BALF samples are widely used for pathogen detection in infectious diseases of the lower respiratory tract because they carry less oral microbial contamination and can yield accurate and representative lung microbial information. Among the 34 BALF samples, the mNGS fastq data of 7 patients was lost due to improper data storage and therefore not within our statistical range, only the remaining 27 patients were further analyzed. Overall, The relative abundance of the top 10 species are shown in Fig. 2A and B. The major species in the non severe *C. psittaci* group were *primate erythroparvovirus 1*, *Candida albicans* and *Cutibacterium acnes*, major species in the severe *C. psittaci* group was *Clavispora lusitaniae*, *Lacticaseibacillus paracasei* and *Candida glabrata*. In addition, further analysis indicated significant differences in the relative Abundance of *Haemophilus parainfluenzae* (Fig. 2C) and *Streptococcus pneumoniae*(Fig. 2D) in the severe and non severe groups. Subsequently, classical Alpha-diversity Shannon and Simpson index analysis and Principal coordinate analysis (PCoA) showed that there was no significant difference in the diversity of lower respiratory tract microbiota between *C. psittac*i pneumonia and severe *C. psittaci* pneumonia(Fig. 2E-G).

### Treatment and outcomes

Treatment and outcomes of patients with *C. psittaci* pneumonia are shown in Table 4. 67.3% patients have a history of empirical antibiotic treatment, Antibiotics that were not active against C. psittaci were administered empirically on admission to the majority of patients with C. psittaci pneumonia. Among these empirical antibiotic treatment patients, 80.43% of patients changed the type of antibacterial drugs according to the results of mNGS. Among the 46 patients, 16 were treated with doxycycline alone, 9 with moxifloxacin alone, 8 with doxycycline and moxifloxacin in combination, 2 with doxycycline and azithromycin in combination, 4 with doxycycline and carbapenems in combination, and 3 with moxifloxacin and carbapenems in combination, another 2 cases were treated with carbapenems. Among 46 patients, 12 patients were treated with invasive mechanical ventilation, 25 patients were treated with nasal catheter, and 3 patients were treated with non-invasive ventilation. Sepsis occurred in 3 patients with severe infection. Finally, 39 patients were cured and discharged, 2 patients gave up treatment, and 3 patients died.The causes of the 3 fatalities were Septic shock, Severe pneumonia and Cardiorespiratory failure.

**Table 4:** Treatment and outcomes of patients with *C. psittaci* pneumonia

Characteristics	*C. psittaci* pneumonia (*n* = 46)	Non severe *C. psittaci* pneumonia (*n* = 19)	Severe *C. psittaci* pneumonia (*n* = 27)	P value
History of empirical treatment	31	11	20	0.2491
**Antimicrobial therapy**				0.1395
Tetracycline	16	9	7	
Quinolones	9	4	5	
Combination therapy	21	6	15	
**Respiratory support, n**				**0.002**
Invasive ventilation	12	0	12	
Non-invasive ventilation	3	0	3	
Oxygen therapy	21	10	7	
High-flow nasal cannula	4	2	6	
No respiratory support	6	6	0	
**Outcomes**				0.1329
Sepsis, n	3	0	3	
Death, n(%)	3	0	3	

### Risk factors associated with severe *C. Psittaci* pneumonia

Multivariate analysis revealed that higher lymphocyte concentrations (OR 0.836, 95% CI 0.714–0.962, *p* = 0.041) were the only protective factor for survival(Table 5).

**Table 5:** Multivariate logistic regression analysis of factors associated with severe *C. psittaci* pneumonia

Variables	Odds ratio	95% confidence interval	P value
Lymphocyte	0.836	0.714–0.962	0.041

## Discussion

In this study, we performed a retrospective analysis of the clinical characteristics of patients with *C. psittaci* pneumonia.The clinical symptoms of these patients were atypical, the major clinical symptoms of *C. psittaci* pneumonia are fever, cough, expectoration. Our results discovered that there was no significant difference in the clinical symptoms between patients with severe *C. psittaci* pneumonia and those non-severe *C. psittaci* pneumonia, which is consistent with a multi-center study of 116 patients with *C. psittaci* pneumonia in Central-South China [21]. But Su et al. reported that the incidence of dyspnea in patients with severe *C. psittaci* pneumonia is higher than that in non severe *C. psittaci* pneumonia patients, and the difference is statistically significant [22]. The majority cases of *C. psittaci* pneumonia have a history of contact with poultry or pigeons, which is also a pivotal clue for the diagnosis of psittacosis. In our study, the main sources of infection were fowl and pigeons, but 14% patients denied any contact history, and the cause of infection remains obscure. Previous studies have investigated that psittacosis can spread among people [23], but fewer than 5% of *C. psittaci* pneumonia cases were community-acquired [23, 24].Our laboratory results indicated that the lymphocyte counts in severe infected patients were lower than that in non severe infected patients, which may reflect a decreased immune function in severe infected patients. In addition, multivariate analysis suggested that higher lymphocyte concentrations were the only survival protective factor for *C. psittaci* pneumonia. Due to the limited number of cases included in this study, whether lymphocyte counts could be used as a predictor of severe disease or death in severe *C. psittaci* pneumonia needs to be further studied [22]. Neutrophils counts, PCT, LDH, HBDH, CK-MB, D-Dimer were higher than that in patients with non severe *C. psittaci* pneumonia, which is consistent with the previous research results of Fang et al [25]. Our results also found that there were 6 patients with severe *C. psittaci* pneumonia complicated with rhabdomyolysis, which represents that it is not uncommon in people with severe *C. psittaci* pneumonia [26].The PCT value in our severe group was higher, indicating mixed infection from multiple pathogens [27]. The mNGS results confirmed the mixed infection findings. In our research, 41.3% (19/46) patients had mixed infection, the coinfections were mostly related to *C. psittaci*, *Acinetobacter baumannii*, *Pseudomonas aeruginosa*, *candida albicans* and *EBV*. Sepsis occurred in 3 patients with severe infection in this study, which may be caused by *Pseudomonas aeruginosa*, *Acinetobacter baumannii*, and *Klebsiella pneumoniae*, respectively. Coinfections in patients with *C. psittaci* pneumonia may lead to more adverse outcomes and need further investigation [28]. two patients accompanied by bacterial infection (both pseudomonas aeruginosa) died. Screening for other respiratory pathogens during the clinical course of severe *C. psittaci* pneumonia is critical for appropriate diagnosis and treatment. Empirical antibiotic treatment should be prescribed for patients with severe *C. psittaci* pneumonia, with rapid de-escalation based on mNGS/culture results [29].

Empirical antimicrobial treatment before being diagnosed with *Chlamydia psittaci* infection is usually ineffective [30]. In our study, 67% of patients underwent empirical antimicrobial treatment, while 80.43% of patients changed their antibiotics after a clear diagnosis was made. The most effective antibacterial drugs for *C. psittaci* pneumonia include quinolones, macrolides and tetracyclines, which are first-line antibiotics for the treatment of psittacosis [31]. In our research, 31 patients were treated with doxycycline and 21 patients were treated with moxifloxacin. In a retrospective study on 116 patients with *C. psittaci* pneumonia, researchers have found that the use of quinolones was related to the reduction of days of hospitalization and days of fever after the use of antibiotics [31]. Additionally, some studies also revealed that the MIC of doxycycline was lower than that of ciprofloxacin in vitro experiments [32]. Due to the difficulty in obtaining doxycycline in China, quinolones are often given as the first choice of drugs. Although some clinical cases reflect the efficacy of quinolones in *C. psittaci* infection, there is still lack of clinical study on the efficacy of quinolones in *C. psittaci* infection at present.

In our study, three patients unfortunately passed away. All three patients underwent tracheal intubation and extracorporeal membrane lung surgery. One of the patients had myelodysplastic syndrome for 10 years, and only one pathogen, *C. psittacii*, was detected through mNGS in this patient, while the other two patients were accompanied by bacterial infections (both Pseudomonas aeruginosa), and clinical testing results showed that all three patients were hypoalbuminemia.Yang M’s research indicated that higher globulin concentration was a protective factor for survival. Respiratory therapy (including high flow nasal intubation, non-invasive ventilation and invasive ventilation) other than oxygen gas was a risk predictor of severe pneumonia [21], Additionally, patients with septic shock and those who require mechanical ventilation are susceptible to secondary infections, which can lead to death [27]. Our results are just in line with this. However, due to the small number of cases, we lack data to verify this statement.

Changes in the diversity or abundance of pulmonary microbiota are related to a variety of chronic respiratory diseases, such as asthma, cystic fibrosis, Bronchiectasis, and chronic obstructive pulmonary disease [28, 33, 34]. The respiratory microbiota may offer resistance to the colonization of respiratory pathogens which also engages in the maturation and maintenance of respiratory physiology and immune homeostasis. The changes in respiratory microbiota may lead to further disease progression and immune imbalance [35]. Therefore, in this study, we investigated the characteristics of the lower respiratory tract microbiota of patients with *C. psittaci* infection by mNGS. The results manifested that there was no statistical difference in the lower respiratory tract microbiota diversity between *C. psittaci* pneumonia and severe *C. psittaci* pneumonia. The microbial species with altered relative abundance in the lower respiratory tract were different in these two groups. Xie et al. found that although reduced pulmonary microecological diversity occurred in both *Chlamydi*a-infected and non-*Chlamydia*-infected patients, the microbial species with altered relative abundance in the lower respiratory tract were significantly different in two patient groups, suggesting that *Chlamydia* infection shapes the characteristics of the pulmonary microbiota in a unique disease state [36]. All these results may provide a possible research direction for unveiling the pathogenic mechanisms of pulmonary infections caused by *Chlamydia*.

Due to the lack of specific clinical manifestations and conventional laboratory tests, the incidence rate of *C. psittaci* pneumonia may be underestimated, Recently, mNGS has emerged as a novel and promising method for the detection of infectious agents. Based on non-preference and high sensitivity, mNGS provides more sensitive pathogen detection results than conventional culture, especially for rare pathogen detection involved in clinically challenging cases. In addition, mNGS has a broad pathogen spectrum, which has the potential to assist clinicians in the diagnosis and treatment of possible mixed infections.

However, There are still several limitations that should be mentioned in this study. First, we only included 46 confirmed patients with *C. psittaci*, and those with strong clinical suspicion but no evidence of the pathogen were excluded, limited research objectives may result in selection bias. Second, as this is a retrospective study, all patients were diagnosed using mNGS and no other laboratory testing methods such as polymerase chain reaction, serological testing, and pathogen culture were used to confirm the diagnosis. Third, The number of cases is not large enough and there is a lack of test results from other conventional testing methods, so it is not possible to verify the sensitivity and specificity of mNGS for detecting *C. psittaci*. we also analyzed lower respiratory tract microbiota characteristics of patients with *C. psittaci* infection by mNGS. Our research still cannot suggest that *C. psittaci* infection disrupts the dynamic balance of the pulmonary microbiome and further impact disease severity. The effects of *C. psittaci* infection on the clinical symptoms and the course of the disease in patients warrant further studies.

## Conclusion

A history of poultry or pigeons exposure could be suggestive of *C. psittaci* pneumonia. *C. psittaci* pneumonia has low clinical incidence and poor clinical specificity, which is easy to cause misdiagnosis and then develop into severe disease, and the overall prognosis is well. Lower respiratory tract infection, especially severe and complex infection, Higher lymphocyte concentrations are protective factors associated with severe *C. psittaci* pneumonia. mNGS has an overall superior detection rate and broader pathogen spectrum than traditional methods and may be particularly useful for concomitant infections. Our findings help to deepen our understanding of the pathogenesis of *C. psittaci* pneumonia, especially Severe *C. psittaci* infections.

## Data Availability

The datasets generated and/or analysed during the current study are available in the National Center Biotechnology Information BioProject database under accession number PRJNA1018096.
