# Correction: Evaluation of clinical characteristics and risk factors associated with Chlamydia psittaci infection based on metagenomic next-generation sequencing

**DOI:** 10.1186/s12866-024-03359-5

**Published:** 2024-06-08

**Authors:** Lei Yuan, Qiang Chen, Xin Yu Zhu, Lan Min Lai, Rui Zhao, Yang Liu

**Affiliations:** Department of Clinical laboratory, The First Affiliated Hospital, Jiangxi Medical College, Nanchang University, No.17, YongWaiZheng Street, Nanchang, 330006 China

**Correction:** ***BMC Microbiol*** **24, 86 (2024)**

10.1186/s12866-024-03236-1

Following publication of the original article [1], the authors would like to correct their affiliation.

The affiliation currently reads:

Department of Clinical laboratory, The First Affiliated Hospital of Nanchang University of Nanchang University, Nanchang University, No.17, YongWaiZheng Street, Nanchang 330,006, China.

The affiliation should read:

Department of Clinical laboratory, The First Affiliated Hospital, Jiangxi Medical College, Nanchang University, No.17, YongWaiZheng Street, Nanchang 330,006, China.
